# Assembly and analysis of the genome of *Notholithocarpus densiflorus*

**DOI:** 10.1093/g3journal/jkae043

**Published:** 2024-03-01

**Authors:** Ying Cai, Ellis Anderson, Wen Xue, Sylvia Wong, Luman Cui, Xiaofang Cheng, Ou Wang, Qing Mao, Sophie Jia Liu, John T Davis, Paulo R Magalang, Douglas Schmidt, Takao Kasuga, Matteo Garbelotto, Radoje Drmanac, Chai-Shian Kua, Charles Cannon, Julin N Maloof, Brock A Peters

**Affiliations:** Advanced Genomics Technology Laboratory, Complete Genomics Inc, San Jose, CA 95134, USA; Advanced Genomics Technology Laboratory, Complete Genomics Inc, San Jose, CA 95134, USA; Advanced Genomics Technology Laboratory, Complete Genomics Inc, San Jose, CA 95134, USA; Advanced Genomics Technology Laboratory, Complete Genomics Inc, San Jose, CA 95134, USA; Department of Research, BGI-Shenzhen, Shenzhen 518083, China; Department of Research, MGI, BGI-Shenzhen, Shenzhen 518083, China; Department of Research, BGI-Shenzhen, Shenzhen 518083, China; Advanced Genomics Technology Laboratory, Complete Genomics Inc, San Jose, CA 95134, USA; Advanced Genomics Technology Laboratory, Complete Genomics Inc, San Jose, CA 95134, USA; Department of Plant Biology, University of California, Davis, CA 95616, USA; Department of Plant Biology, University of California, Davis, CA 95616, USA; Department of Environmental Science, Policy and Management, University of California, Berkeley, CA 94720, USA; Crops Pathology and Genetics Research Unit, United States Department of Agriculture—Agricultural Research Service, Davis, CA 95616, USA; Department of Environmental Science, Policy and Management, University of California, Berkeley, CA 94720, USA; Advanced Genomics Technology Laboratory, Complete Genomics Inc, San Jose, CA 95134, USA; Center for Tree Science, The Morton Arboretum, Lisle, IL 60532, USA; Center for Tree Science, The Morton Arboretum, Lisle, IL 60532, USA; Department of Plant Biology, University of California, Davis, CA 95616, USA; Advanced Genomics Technology Laboratory, Complete Genomics Inc, San Jose, CA 95134, USA

**Keywords:** stLFR, cobarcoding, tanoak, SOD

## Abstract

Tanoak (*Notholithocarpus densiflorus*) is an evergreen tree in the Fagaceae family found in California and southern Oregon. Historically, tanoak acorns were an important food source for Native American tribes, and the bark was used extensively in the leather tanning process. Long considered a disjunct relictual element of the Asian stone oaks (*Lithocarpus* spp.), phylogenetic analysis has determined that the tanoak is an example of convergent evolution. Tanoaks are deeply divergent from oaks (*Quercus*) of the Pacific Northwest and comprise a new genus with a single species. These trees are highly susceptible to “sudden oak death” (SOD), a plant pathogen (*Phytophthora ramorum*) that has caused widespread deaths of tanoaks. In this study, we set out to assemble the genome and perform comparative studies among a number of individuals that demonstrated varying levels of susceptibility to SOD. First, we sequenced and de novo assembled a draft reference genome of *N. densiflorus* using cobarcoded library processing methods and an MGI DNBSEQ-G400 sequencer. To increase the contiguity of the final assembly, we also sequenced Oxford Nanopore long reads to 30× coverage. To our knowledge, the draft genome reported here is one of the more contiguous and complete genomes of a tree species published to date, with a contig N50 of ∼1.2 Mb, a scaffold N50 of ∼2.1 Mb, and a complete gene score of 95.5% through BUSCO analysis. In addition, we sequenced 11 genetically distinct individuals and mapped these onto the draft reference genome, enabling the discovery of almost 25 million single nucleotide polymorphisms and ∼4.4 million small insertions and deletions. Finally, using cobarcoded data, we were able to generate a complete haplotype coverage of all 11 genomes.

## Introduction

Tanoak (*Notholithocarpus densiflorus*; Manos *et al*. 2008) is part of the beech family (Fagaceae) and possesses an unusual evolutionary history. Long considered a disjunct relictual element of the Asian stone oaks (*Lithocarpus*), modern phylogenetic analysis determines that the tanoak is a clear example of convergent evolution in fruit type, requiring the recognition of a new genus comprising a single species (Manos *et al*. 2008). More recent phylogenomic analyses (Zhou *et al*. 2022) place it basal and sister to all northern hemisphere oaks (genus *Quercus*), both Old and New World groups. Tanoak is also the last common ancestor with insect pollination in a species-rich wind-pollinated clade, splitting with the oaks roughly 54 million years ago. Little fossil evidence for the taxon exists, but its current geographic distribution is restricted to a relatively small area in the Pacific Northwest, suggesting that this taxon may have belonged to a species-poor clade for a significant period of time. By comparison, the North American oaks have diversified and spread throughout North America, occupying a wide range of habitats (Hipp *et al*. 2018).

Ecologically, tanoaks are adapted to a Mediterranean-type climate, with a long dry season and periodic fires. They can tolerate a wide range of soil types, from shallow rocky soils to deep, well-drained soils. Two growth forms exist, recognized as different varieties: *N. densiflorus* var. *densiflorus* is a tree, with individuals growing to 45 m in height, often as a codominant in the redwood and mixed evergreen forests of the north coast ranges, while *N. densiflorus* var. *echinoides* is a shrub, more commonly growing at higher elevations in open conifer forests and dry slopes of the northern interior. As a locally dominant species in these habitats, *Notholithocarpus* trees play an important ecosystem role, forming the mid and lower canopy strata of redwood forests and providing habitat and food for a variety of wildlife, including birds and mammals (Waring and O’Hara 2008). Additionally, their thick bark and ability to resprout from the base, after fire or other damage, make them an important component of the forest's resilience and recovery.

Tanoak obtained its common name from the extensive harvest of their bark during the early 20th century for the regional tanning industry (Bowcutt 2011), a business that paradoxically first led to increased tanoak densities due to prolific coppicing and then made it a frequent target of herbicide applications to reduce densities. Both the acorns and the bark of Notholithocarpus trees have been used for food and leather processing by indigenous peoples in North America for centuries (Bowcutt 2015). Sudden oak death (SOD), caused by the oomycete pathogen, *Phytophthora ramorum*, has killed tens of millions of tanoak, coast live oak, California black oak, and other native tree species (Aphis.usda.gov). Tanoak is the most susceptible species to *P. ramorum* (Davidson *et al*. 2003). Its decline due to the rapid spread of SOD has the potential to dramatically affect the overall biodiversity and conservation status of these forests (Cobb *et al*. 2012), particularly compromising their role as one of the few local ectomycorrhizal hosts (Bergemann and Garbelotto 2006). Overall, the loss of tanoak from redwood forests will reduce biodiversity and alter fundamental ecosystem processes (McCallum and Dobson 1995; Rizzo *et al*. 2005; Wardle *et al*. 2011).

The difference in the susceptibility to SOD between Notholithocarpus and Quercus is also a compelling question, potentially associated with the dramatic differences in their evolutionary history and reproductive biology. North American oak species generally participate in a large continental scale syngameon (Cannon *et al*. in review), which potentially enhances the overall diversity found in their genome and particularly in disease-resistance genes (Cannon and Petit 2020). This genetic exchange among oak species is probably facilitated by their wind pollination, in comparison with the insect-pollinated tanoak. The existence of a single species of tanoak obviously prevents it from gaining any evolutionary advantage from participation in a syngameon, regardless of its pollination syndrome. This substantial difference between these 2 approximately similar-aged lineages—the species-rich syngameon of the oaks vs the species-poor (monospecific) isolate of the tanoak—should have a considerable impact on the overall genomic evolution and potential susceptibility to SOD. In this study, we set out to assemble the genome of tanoak and perform comparative studies among a number of individuals that demonstrated varying levels of susceptibility to SOD. We then compared this assembly with existing completed genomes in the *Fagaceae*. The questions we asked were as follows:

1. Do basic genomic properties differ between species-rich and species-poor lineages?
2. Can differences in the overall diversity of disease-resistance genes be detected?

## Materials and methods

### DNA isolation

For each sample, a single whole leaf was placed in an Oster Pro 1200 blender with 100 ml of lysis buffer [13 mM Tris-HCl (pH 8.3), 140 mM NaCl, 3 mM KCl, 350 mM sucrose, 1 mM EDTA, and 1% Triton X-100] and blended on high for 5 min. Lysates were pelleted at 2,900 × *g* for 15 min. Supernatants were discarded, and the pellet was further isolated using a Nanobind Plant Nuclei Big DNA kit (Circulomics, Baltimore, MD, USA) following the manufacturer's protocol. Samples were incubated with proteinase K for 2 h, eluted in 100 μl of elution buffer, and quantified using a Nanodrop 1000 spectrophotometer (ThermoFisher, Waltham, MA, USA). To enrich for longer DNA molecules, six samples were further processed using a Short Read Eliminator XL kit (Circulomics) prior to making single-tube long fragment read (stLFR) libraries. This long fragment-enriched DNA was also used for the Minion sequencing (Oxford Nanopore Technologies, Oxford, UK).

### Cobarcoded read libraries

Cobarcoded read libraries were generated using an MGIEasy stLFR Library Prep kit (MGI, Shenzhen, China) following the manufacturer's protocol using 1 ng of input DNA. stLFR libraries were analyzed on a DNBSEQ-G400 (MGI) DNA sequencer using pair-end 100 base reads and a 42-base barcode read. stLFR fq files were processed using the barcode split tool (GitHub; https://github.com/stLFR/stLFR_read_demux; Wang *et al*. 2019) to deconvolute barcodes.

### Nanopore libraries

Minion libraries were prepared using the Genomic DNA by Ligation kit (Oxford Nanopore Technologies) following the recommended protocol. Briefly, the isolated DNA was first repaired and end-preprepared by the NEBNext FFPE DNA Repair mix (New England Biolabs, Ipswich, MA, USA) and the NEBNext Ultra II End repair/dA-tailing Module (New England Biolabs) following the manufacturer's protocol. The reaction was purified using a 1× volume of Ampure XP beads (Beckman Coulter, A63882) following the manufacturer's protocol. The product was then ligated with the Adapter Mix and purified with an optimized protocol provided by the Genomic DNA by Ligation kit. After purification, the library was ready for sequencing.

Minion sequencing was carried out following the manufacturer's suggested protocol. The priming buffer mix was first prepared in accordance with the protocol and then loaded onto an R9.4.1 flow cell. The final sequencing library was prepared by mixing 50 fmol of a purified library with the sequencing buffer and the loading beads. The loaded flow cell was then mounted onto a MinION Mk 1B device (Oxford Nanopore Technologies) and sequenced with MinKNOW v19.10 for ∼24 h. FAST5 files were analyzed with Guppy and configuration file dna_r9.4.1_450bps_fast.cfg.

### Genome assembly

Cobarcoded sequencing reads from stLFR data were assembled using a modified version of Supernova (10X Genomics, Pleasanton, CA, USA) that allows for >4 million unique barcodes. The 6 Supernova assemblies generated from DNA enriched for long fragments were used to build a single genome assembly for tanoak by using contigs from NL.2.XL, SM.52.81.XL (clone 2), LP.22.48.XL, SM.52.42.XL, and SM.54.37.XL to fill gaps in the SM.74.45.XL (clone 1) assembly. This was performed using a TGS-gapfiller with standard settings. To further improve the pangenome assembly, TGS-gapfiller (Xu *et al*. 2020) was used with 11.8, 9.6, and 6 Gb of Minion-generated reads from SM.54.37.XL, SM.52.82.XL (clone 2), and NL.2.XL, respectively. Finally, SM.74.45.XL (clone 1) cobarcoded reads were aligned to the genome assembly with bwa, and the genome was polished using pilon.

We observed 2 possible misassemblies on the dotplot [Oxford Nanopore (ONT) contig_83 aligned to draft genome on both 262_pilon and 656_pilon; contig_513 aligned to 360_pilon and 819_pilon]. We aligned the ONT reads to both the ONT assembly and the draft genome but found no conclusive evidence to indicate which assembly was the correct one. Future investigation with improved sequencing techniques will be necessary to disambiguate this region.

After performing a joint calling of all 19 sequencing libraries (see the “Variant calling and phasing” section), we discovered 58,314 homozygous alternative allele variants shared by all 19 libraries. This suggested that these alternative alleles should, in fact, be the reference allele. To correct this, we replaced all 58,134 positions in the reference with the alternative allele and created a new v2 reference. For most analyses, this change was immaterial, and v1 was continued to be used, but for all variant calling applications, v2 was used.

### Genome analysis

Genome completeness and contiguity was analyzed with BUSCO version 5.2.2 (Manni *et al*. 2021) using standard options with the embryophyta_odb10 dataset. N50 statistics and other genome metrics were generated using QUAST version 5.0.2 with default settings.

The draft genome was aligned to *Quercus robur* and *Q. rubra* genomes with minimap2 v.2.16-r922 (Li 2018). The alignment results were then visualized with pafCoordsDotPlotly.R from dotPlotly (https://github.com/tpoorten/dotPlotly).

### Genome annotation

Protein-coding gene annotation was performed using MAKER v 3.01.04 (Cantarel *et al*. 2008; Campbell *et al*. 2014). A de novo transcriptome assembly, protein sequences from 2 related oak species (Plomion *et al*. 2018), a tanoak repetitive elements library, and a tanoak gene prediction model were used as gene evidence for the initial round of gene prediction with default parameters. The de novo transcriptome was assembled by Trinity v2.8.5 (Grabherr *et al*. 2011) with input mRNA from an SRA study SRP157197 (Kasuga *et al*. 2021) of 45 samples. Protein sequences emanated from *Q. robur* (English oak) and *Q. rubra* (northern red oak). The repeat library was established using RepeatMasker v4.0.7 (A.F.A. Smit, R. Hubley & P. Green RepeatMasker at http://repeatmasker.org) following the MAKERP pipeline method (Campbell *et al*. 2014). The gene prediction model was created using the BUSCO v4.1.4 (Manni *et al*. 2021) pipeline with AUGUSTUS (Stanke and Waack 2003) in genome mode with the lineage eukaryota_odb10. The resulting gene models from MAKER were then used to train SNAP (Korf 2004) and create an HMM file. MAKER was then used for a second round of gene prediction employing the previously mentioned gene evidence along with the first round MAKER annotations and the SNAP HMM file. The resulting gene models were then filtered to keep annotations with an annotation edit distance (AED) ≤ 0.5.

The MAKER-generated proteins were compared against the UniProt/SwissProt database (UniProt 2021) with BLASTP (BLAST v2.13.0+) to obtain a homology-based annotation. Interproscan v5.59.91.0 was used to identify protein domains and predicted Gene Ontology (GO) terms. A total of 51,233 protein-coding genes were identified.

### Variant calling and phasing

The Genome Analysis ToolKit (v.4.1.2.0) was used for variant calling. For each sample, the HaplotypeCaller function was used to call GVCF files. After combining all GVCF files, the GenotypeGVCFs function was used to join genotype variants. The variants were hard-filtered to keep ≥15× coverage across all samples. Low-quality variants were removed with QD < 2.0 || MQ < 26.0 || FS > 100.0 || SOR > 5.0 || MQRankSum < −7.5 || ReadPosRankSum < −8.0 [parameters adopted from (Hu *et al*. 2022)]. The resulting high-quality variants were phased with Hapcut2 v.1.3 (Bansal 2023) for each sample. Due to high diversity and variant calling errors, 2 haplotypes are considered the same if they share >90% similarity of single nucleotide polymorphism (SNP) calls.

### Polymorphism analyses

Scripts for the SnpEff annotation, the *Arabidopsis* annotation, principal component analysis (PCA), and nonsynonymous to synonymous substitutions (*dN*/*dS*) analyses described below are available at https://github.com/MaloofLab/Cai-TanOak-2024. SnpEff v5.1d (Cingolani *et al*. 2012) was used to predict the possible consequences of each SNP on protein-coding genes. Filtering and analysis of SNPs was performed using custom scripts in R (R Core Team 2021) and the R and Bioconductor packages VariantAnnotation (Obenchain *et al*. 2014), ggplot2 (Wickham 2016), and tidyverse (Wickham *et al*. 2019). To determine the closest *Arabidopsis* homolog for each tanoak gene, blastp (Altschul *et al*. 1990, 1997) was used to blast the tanoak proteome against Arabidopsis TAIR10 protein sequences (downloaded from https://arabidopsis.org/download_files/Genes/TAIR10_genome_release/TAIR10_blastsets/TAIR10_pep_20110103_representative_gene_model_updated). Additional functional annotation was performed using interproscan 5.30-69.0 (Mitchell *et al*. 2019). For *dN*/*dS* analysis, tanoak/oak (*Q. robur*) homologs were identified using blastp (Altschul *et al*. 1990, 1997). Orthologs were then defined as gene pairs with reciprocal blastp best hits, *e*-values <1e−04, where the next best hit had an e-value at least 100 times greater than the candidate ortholog. Ortholog pairs were aligned using MASCE v2.05 (Ranwez *et al*. 2018), and *dN*/*dS* was calculated using SeqinR (Charif and Lobry 2007). Ortholog pairs were binned according to their *dN*/*dS* value, and Fisher's exact test was used to determine GO enrichment among the *dN*/*dS* bins.

## Results and discussion

**Fig. 1.**
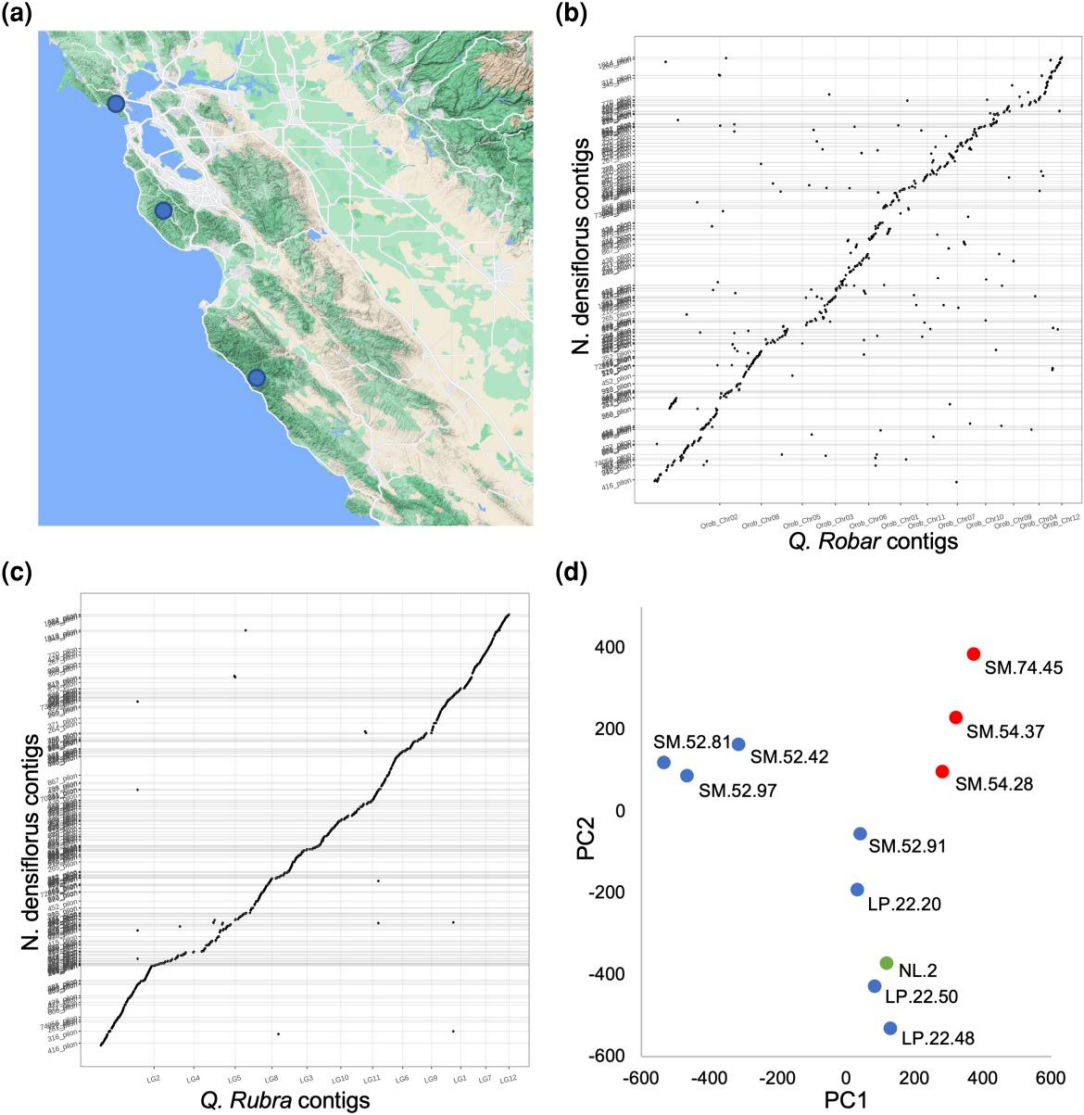
*N. densiflorus* project characteristics. a) Samples were collected in multiple locations across Central California as displayed on the map (*Map data ©2021 Google).* Assembled contigs from the draft tanoak reference (*y*-axis) were compared against the *Q. robur* b) and *Q. rubra* c) assemblies (*x*-axis). d) The 11 genetically distinct tanoak samples were projected onto a PCA generated from 2.4 million bi-allelic SNPs. The first (*x*-axis) and second (*y*-axis) principal components were plotted for each sample. Tree specimens SM.74.45, SM.54.37, and SM.54.28 have shown increased susceptibility to *P. ramorum.* The remaining samples, apart from NL-2 which has not been measured, have shown varying degrees of resistance to *P. ramorum*.

Thirteen individual trees, 11 of which are genetically distinct and from disparate locations, were selected in order to help gauge the diversity within the *N. densiflorus* species (Fig. 1a). In addition, trees from the University of California Long-Term Tanoak Orchard were selected based on various levels of susceptibility to *P. ramorum*, the plant pathogen that causes SOD, ranging from relatively resistant to highly susceptible (Hayden *et al*. 2011; Supplementary Table 1). Genomic DNA from a leaf of each tree was used to make cobarcoded sequencing libraries using the stLFR process (Cheng *et al*. 2018; Wang *et al*. 2019). Approximately 100 Gb of data per sample were generated using an MGI DNBSEQ-G400 second-generation DNA sequencer (Supplementary Table 1). Reads from each sample were analyzed with GenomScope (Vurture *et al*. 2017) to determine the kmer spectra and the heterogeneity of each sample as well as estimate the size of the *N. densiflorus* genome (Supplementary Table 2 and Fig. 1). Both the estimated size and the kmer heterogeneity fell within the range of other closely related species (Supplementary Table 3).

The genomic DNA of 6 samples was further enriched for higher molecular weight fragments (labeled with XL), and additional stLFR libraries were made and processed as above but with ∼200 Gb of data generated per sample. These were individually de novo diploid assembled using a modified version of 10X Genomics’ Supernova software (Weisenfeld *et al*. 2017) resulting in contig and scaffold N50 values ranging from 31.4 to 50.4 kb and 0.145 to 2.05 Mb, respectively (Supplementary Table 4). Using Merqury (Rhie *et al*. 2020), a kmer based assembly analysis program, a per base quality score ranging from Q50 to 59, and an estimated completeness of 82–89% (Supplementary Table 4) were generated for each of the 6 samples. A single pseudo haplotype with the overall best assembly (contig N50 of 50.4 kb, scaffold N50 of 2.05 Mb, 89% complete, and Q59) was selected [SM.74.45.XL (clone 1)] for use as the draft reference *N. densiflorus* genome, and contigs from the remaining 5 XL assemblies were used to fill gaps within each scaffold of the draft using TGS-GapCloser (Xu *et al*. 2020). This resulted in a large improvement in contiguity from an N50 of 50.4 to 385.8 kb (Table 1). To further increase contiguity, 27 Gb of nanopore data (ONT) from NL2.XL, SM.52.81.XL (clone 2), and SM.54.37XL were used to fill the remaining gaps and achieve a contig N50 of ∼1 Mb (Table 1). This assembly was further polished to remove errors using Pilon (Walker *et al*. 2014) with the NGS read set from SM.74.45.XL (clone 1). Purge Haplotigs (Roach *et al*. 2018) was used to remove duplicated regions in the genome and resulted in a reduction in size from 916.6 to 777.5 Mb. This is closer to the expected size of 785 Mb as determined by flow cytometry (Supplementary Fig. 2).

**Table 1.** *N. densiflorus* genome assembly statistics.

	SM.74.45.XL (clone 1) hap1	Gapped filled with assembled contigs	Gapped filled with ONT reads	Final draft reference genome
Assembly statistics				
Contig N50 (kb)	40.8	385.8	1,034.5	1,221.7
Number of contigs	37,819	23,251	21,545	11,978
Scaffold N50 (Mb)	1.5	1.7	1.7	2.1
Number of scaffolds	21,136	21,183	21,216	11,387
Assembly size (Mb)	840.5	880.1	884.0	777.5
Ns per 100 kb	8,693	1,525	653	716
BUSCO analysis				
Complete (single copy)	95.3% (87.1%)	95.2% (85.8%)	95.6% (86.1%)	95.5% (91.0%)
Fragmented	2.90%	3.00%	2.60%	2.60%
Missing	1.80%	1.80%	1.80%	1.90%

In addition, the entire set of ONT reads was assembled using Flye v2.9 (Kolmogorov *et al*. 2019). This assembly was aligned with the draft reference in order to find potential insertions and deletion errors (Supplementary Fig. 3). Overall, 2 assemblies aligned very closely with only one large region found to be duplicated in the draft reference vs the ONT assembly. Further inspection of read coverage in this region, after mapping all of the samples to the draft reference, suggested that this duplication is present in the tanoak genome and should not be removed. In each case, raw ONT reads were used to determine what corrective actions should be taken. Finally, BUSCO (Manni *et al*. 2021) analysis was performed on the draft genome resulting in a complete gene score of 95.5% with a low duplication rate of 4.5% (Table 1). In addition, dot plots between the tanoak draft genome and 2 related species of oaks (*Q. robur* and *Q. rubra*; Fig. 1b and c) showed close alignment. Taken together, these results suggest that we have generated a high-quality draft reference genome for *N. densiflorus.*

Next, we proceeded to annotate the coding sequences of the draft genome with MAKER (Campbell *et al*. 2014), AUGUSTUS (Stanke and Waack 2003), SNAP (Korf 2004), protein sequence from the taxonomically close *Q. robur* (Plomion *et al*. 2018), and a Trinity (Grabherr *et al*. 2011) de novo assembled transcriptome from a study of RNA-sequencing data from 45 different *N. densiflorus* samples (Kasuga *et al*. 2021). This resulted in the placement of 42,319 genes onto the draft genome (Table 2). To explore the resistance (R) gene content [nucleotide-binding site leucine-rich repeat (NLR) genes], we used an NLR annotator (Steuernagel *et al*. 2020) and compared the tanoak results with the published genomes of 9 different plant species (Table 3). *Q. robur* has been found to be more resistant to *P. ramorum* and was found to have ∼1.6-fold more complete nonpseudo R genes than *N. densiflorus* (Table 3). While intriguing, further studies will be necessary to determine the cause of increased resistance in *Q. robur*.

**Table 2.** Gene annotation and repeat element summary statistics for *N. densiflorus*.

Category	Total bases (Mb)	% of genome	Mean length	Median length
Genes	169	21.7	3,990	2,495
Coding Sequence (CDS)	44	5.7	1,039	804
Repeats	364	46.8		

**Table 3.** *N. densiflorus* R gene content vs other species.

Species	TNL*^a^*	CNL*^b^*	Total	Complete	Complete pseudo	Partial	Partial pseudo	Genome size (Mb)	Complete/genome Size	Reference
Walnut (*Juglans regia*)	199	145	460	251	129	27	53	573	0.44	Peng *et al*. (2017)
Chinese chestnut (*Castanea mollissima*)	143	188	418	242	115	31	30	413	0.59	Staton *et al*. (2020)
Tanoak (*N. densiflorus*)	302	398	947	505	248	126	68	778	0.65	This study
Poplar (*Populus trichocarpa*)	213	204	637	359	143	77	58	434	0.83	Tuskan *et al*. (2006)
Northern red oak (*Q. rubra*)	354	510	1320	613	327	77	88	740	0.83	Kapoor *et al*. (2023)
Grape (*Vitis vinifera*)	174	336	739	416	202	74	47	486	0.86	Jaillon *et al*. (2007)
Pendunculate oak (*Q. robur*)	494	554	1319	773	360	102	84	814	0.95	Plomion *et al*. (2018)
*Arabidopsis thaliana*	115	30	171	122	20	21	8	120	1.02	Lamesch *et al*. (2012)
Peach (*Prunus persica*)	166	186	415	257	92	37	29	227	1.13	International Peach Genome Initiative *et al*. (2013)
European beech (*Fagus sylvatica*)	376	547	1,290	699	375	112	104	541	1.29	Mishra *et al*. (2021)

^*a*^Toll/interleukin-1 receptor-nucleotide binding site-leucine rich repeat domain containing.

^*b*^Coiled coil-nucleotide binding site-leucine rich repeat domain containing.

**Fig. 2.**
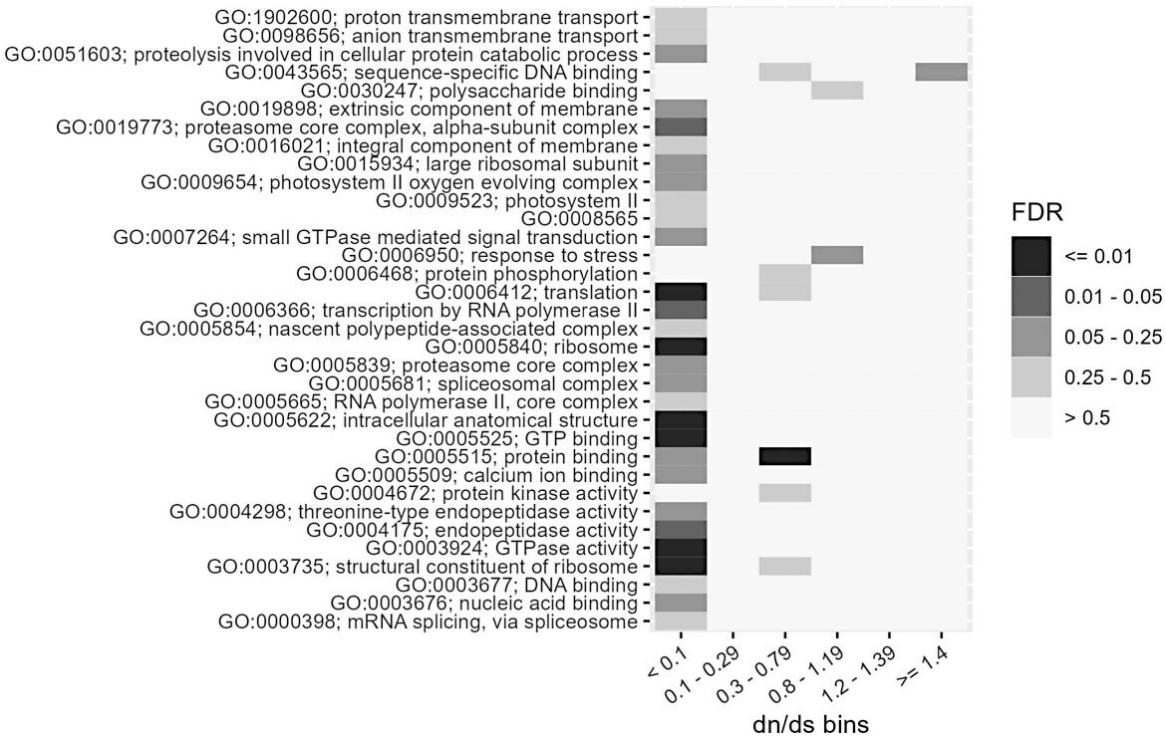
A *dN*/*dS* analysis. Genes were binned based on their *dN*/*dS* ratio (*x*-axis) and then GO enrichment was performed for genes in each bin. The shading indicates the false discovery rate for GO enrichment.

To look for genes that may have been subject to selection since the divergence of oak and tanoak, we calculated the ratio of *dN*/*dS* for all annotated tanoak genes for which we could identify a clear oak ortholog by the reciprocal blast. A total of 5,541 were found to have a clear ortholog, GO annotation, and sequence variation between these species. To ask whether particular types of genes were enriched in genes showing signs of purifying or positive selection, we binned genes based on their *dN*/*dS* value and calculated GO enrichment for each bin (Fig. 2). Three hundred fifty-nine genes showed evidence of strong purifying selection (*dN*/*dS* < 0.1) in processes such as protein translation, ribosomes, protein degradation, and RNA pol II transcription, as expected based on the fundamental nature of these processes. With regard to positive selection, 201 genes were found to have a *dN*/*dS* ratio above 1.2, and the GO term “sequence-specific DNA binding” was marginally enriched for genes with a *dN*/*dS* ≥1.4 (false discovery rate [FDR] = 0.20). Interestingly, 8 of 9 genes in this category had homology to *Arabidopsis* genes related to pathogen defense or abiotic stress (the 9th gene did not have a functionally annotated homolog; Supplementary Table 5). Specifically, there were 3 genes with homology to *Arabidopsis* WRKY transcription factors each implicated in microbial defense (WRKY 11, 40, and 41), 3 genes with homology to *Arabidopsis* genes regulated by abscisic acid (RAS1 and 2 ATHB7 homologs), and 2 genes with homology to *Arabidopsis* heat stress transcription factors (AtHSFA-2 and 3).

In order to investigate the variation between samples, we mapped all of the individual libraries onto the newly created reference genome. This resulted in the discovery of ∼25 million SNPs and of ∼4.4 million small insertions and deletions with an average of 7.7 million per individual tree. PCA was performed using SNP data from all libraries (Fig. 1d and Supplementary Fig. 4). As expected, replicate libraries made from the same DNA sample and libraries from related family members tended to cluster with each other (Supplementary Fig. 4). Projecting SOD susceptibility on the PCA did not inform beyond what was already known based on family inheritance (Fig. 1d).

**Fig. 3.**
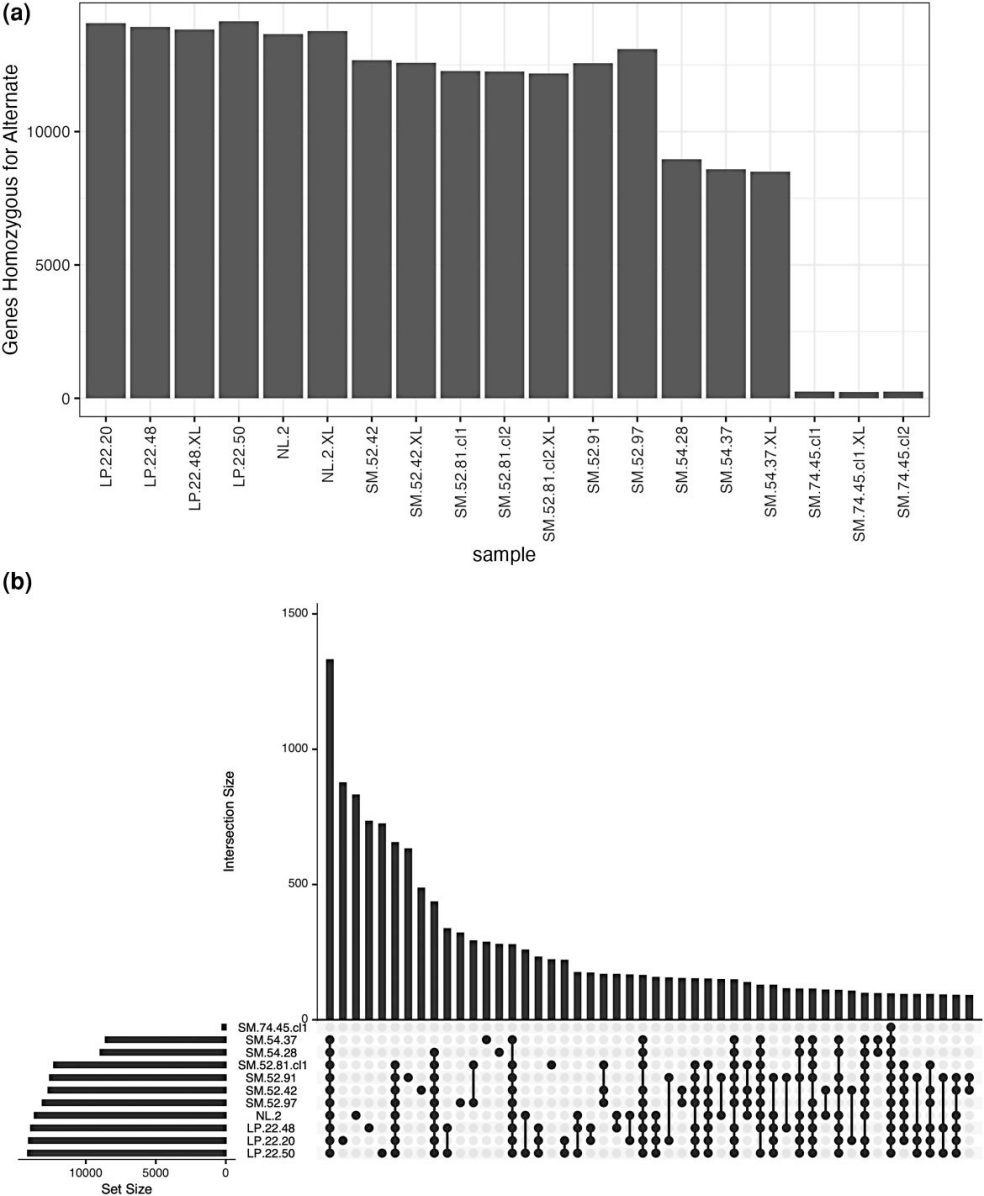
The distribution of moderate- and high-effect alleles. a) The number of genes with at least one moderate- or high-effect alternate allele (compared with SM.74.45 reference) for each sample. b) An UpSet plot (Lex *et al*. 2014; Conway *et al*. 2017) showing the unique and shared alleles among samples.

Of the total variants identified in tanoak, 604,032 resulted in coding changes to 39,574 different genes. Using SnpEff (Cingolani *et al*. 2012), these were further evaluated resulting in the categorization of 526,584 SNPs predicted to have a moderate impact on 38,837 genes and 77,687 SNPs with a high-impact on 22,361 genes (239 SNPs are predicted to have a high impact on one gene and a moderate impact on another gene [for example transcribed from the opposite strand], therefore the total number of SNPs categorized as high and moderate impact is slightly higher than the total number of coding SNPs). Comparing the reference tree SM.74.45 with the other trees, we found that SM.54.28 and SM.54.37 differ from the reference at high and/or moderate alleles in 8,499–8,965 genes, and the remaining trees differed from the reference in 13,095–14,069 genes (Fig. 3a). We also compared the overlap of alternate alleles using an UpSet plot (Lex *et al*. 2014; Conway *et al*. 2017; Fig. 3b). Unsurprisingly, the most common categories are those SNPs that are alternate in all trees except for the reference. Interestingly, the next 4 most common categories are SNPs that are unique to individual trees (or trees and clones), indicating a high degree of diversity among these trees. This plot reveals that each tree has a large number of unique SNPs. Additionally, when all libraries are added to this plot (Supplementary Fig. 5), the concordance across clones and “XL” samples of the same tree are shown.

**Fig. 4.**
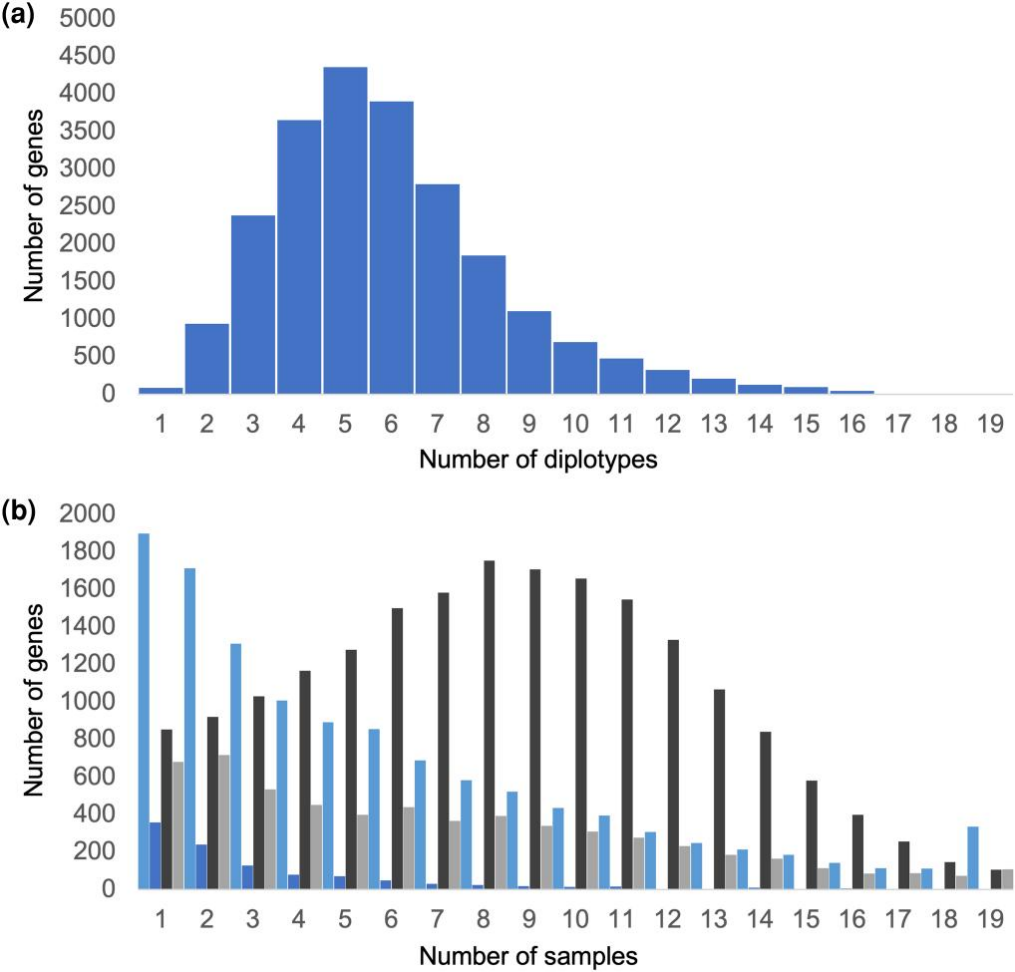
A gene diplotype analysis. The total number of gene diplotypes across the entire set of samples were calculated using haplotype information from each sample. a) Diplotypes per gene were calculated and summed resulting in a median of 6. b) The number of samples with at least one moderate (light blue) or high (blue) SnpEff called variant in each allele of a specific gene was summed. The same calculations were done for 2 or more moderate (dark gray) or high (gray) SnpEff called variants in the same allele of a specific gene.

One of the unique advantages of using stLFR to analyze these samples is that genome-wide haplotype data could be generated for all samples. Using HapCut2, an average haplotype contig N50 value of ∼1.6 Mb was achieved enabling the exploration of haplotype variation across different samples (Supplementary Fig. 6 and Table 1) and enabling the determination of 136,541 diplotypes (a specific combination of 2 individual haplotypes) across 23,089 genes, with a median of 6 diplotypes per gene (Fig. 4a). Combining this information with the SnpEff analysis allowed the discovery of 188 genes on average per tree with moderate- or high-impact changes predicted in both alleles (Fig. 4b).

### Conclusions

In this study, we sequenced and de novo assembled a draft reference genome for the species *N. densiflorus*, a member of the beech family, using cobarcoded second-generation reads. Using kmer analysis, we estimated that the initial assembly had ∼1 error in 850,000 bases (Q59.3). We further refined and filled gaps in this assembly by adding contigs from other assembled tanoak samples as well as through the use of third-generation continuous reads. The draft reference we presented in this study is one of the most contiguous tree genomes available with contig and scaffold N50s of ∼1.2 and ∼2.1 Mb, respectively. BUSCO analysis, as well as the alignment of this reference to other closely related species and to an assembly of tanoak using only third-generation reads, suggests that the tanoak draft reference is assembled accurately. Using transcriptome and in silico data, we identified and placed 42,331 genes on the draft reference. In addition, we sequenced a total of 11 unique tanoak trees to better understand the intraspecific diversity. The advanced features of cobarcoded sequencing reads also enabled us to generate haplotype information for each sample with an average N50 of ∼1.6 Mb.

A comparison of the tanoak genome, which has evolved as a species-poor lineage with a limited geographic distribution for a significant period of time, with other related tree genomes (Supplmentary Table 3) showed a similar amount of heterogeneity, which is surprising given the complexity of the evolutionary history of oaks (*Quercus*), which has involved substantial introgression within a species-rich syngameon (Hipp *et al.* 2020). Interestingly, the analysis of the R gene composition of tanoak vs other tree genomes showed that tanoaks had an overall lower number than many related species, and when taking genome size into account, the ratio of R genes to genome size was one of the smallest we measured, particularly among its close relatives in the Fagaceae (Table 3). We would suggest this low number of R genes may be due to its long-term evolutionary isolation and lack of participation in a larger syngameon (Cannon and Petit 2020), as the oaks do, where adaptive introgression can restore and enrich positively selected gene families. A *dN*/*dS* comparison of tanoak with pendunculate oak revealed some positive selection in tanoak for pathogen defense and abiotic stress genes (Plomion *et al*. 2018), although it was unclear what phenotypic impact this positive selection would have on tanoak. With the rapid increase in available high-quality genomic assemblies in the Fagaceae, further comparative studies will help elucidate the long-term genomic impacts of participation on a species-rich syngameon vs a single reproductively isolated species.

Using this draft reference, we explored the diversity between different tree samples and identified coding changes that potentially may result in severe alteration of protein function in over 22,000 genes. Comparing this information with SOD resistance in each sample resulted in the discovery of some interesting genes involved in plant immunity and signaling, but none reached statistical significance, and further studies will be needed to understand individual tree resistance to *P. ramorum.*

Finally, the unique cobarcoding sequencing method we used enabled the ordering of variants into long haplotypes covering the majority of the genome of each sample. This information resulted in the determination of over 136,000 unique combinations of potentially expressed genes. In all, this reference and the additional samples sequenced provided a glimpse into the inner workings of tanoak and also some sense of the diversity across this species. We hope this reference will be of help to researchers studying tanoak, especially those working to find ways to improve the health and survival of this important species.

## Supplementary Material

jkae043_Supplementary_Data

## Data Availability

Cobarcoded and ONT sequencing data generated for this study have been deposited in the SRA under BioProject PRJNA944640. The *Notholithocarpus densiflorus* draft assembly has been deposited at DDBJ/ENA/GenBank under the accession JARYZH000000000. For most analyses, the version described in this paper is JARYZH000000000.1. For variant calling, an updated version JARYZH000000000.2 was generated that corrected alternative homozygous variants found in all samples to be the reference bases. Scripts for the SNP association tests, SnpEff annotation, *Arabidopsis* annotation, PCA, and *dN*/*dS* analyses are available at https://github.com/MaloofLab/Cai-TanOak-2024. Supplemental material available at G3 online.
