# Supplementary material for: Assembly and analysis of the genome of Notholithocarpus densiflorus
Source: G3 (Bethesda). 2024 Mar 1;14(5):jkae043. doi: 10.1093/g3journal/jkae043 (PMC11075539; doi:10.1093/g3journal/jkae043)
Supplement: jkae043_Supplementary_Data [file jkae043_supplementary_data.zip › Supplemental_Figures_G3-2023-404749.pptx]

## Slide 1
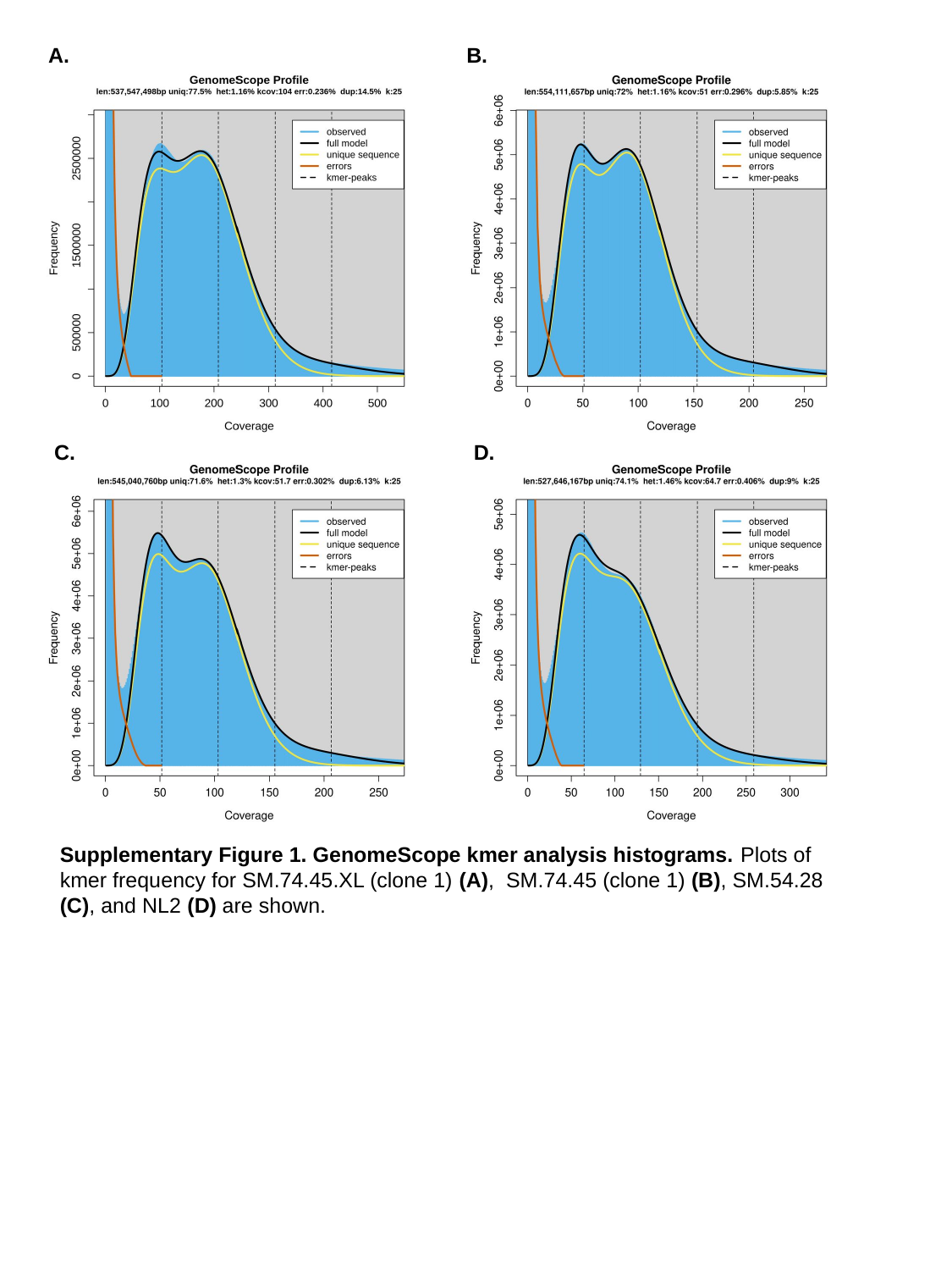

A.
B.
C.
D.
Supplementary Figure 1. GenomeScope kmer analysis histograms. Plots of kmer frequency for SM.74.45.XL (clone 1) (A),  SM.74.45 (clone 1) (B), SM.54.28 (C), and NL2 (D) are shown.

## Slide 2
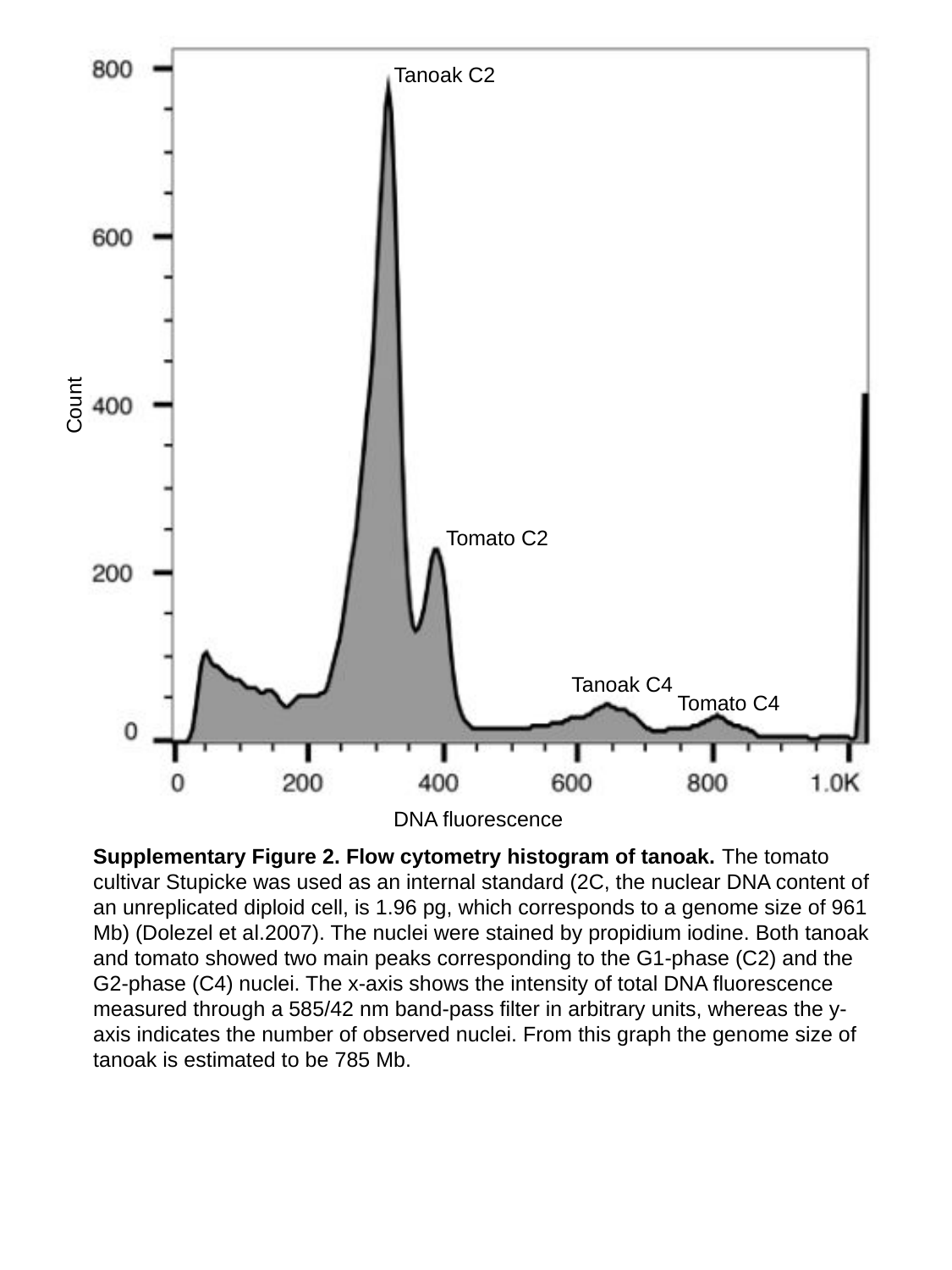

Tanoak C2
Count
Tomato C2
Tanoak C4
Tomato C4
DNA fluorescence
Supplementary Figure 2. Flow cytometry histogram of tanoak. The tomato cultivar Stupicke was used as an internal standard (2C, the nuclear DNA content of an unreplicated diploid cell, is 1.96 pg, which corresponds to a genome size of 961 Mb) (Dolezel et al.2007). The nuclei were stained by propidium iodine. Both tanoak and tomato showed two main peaks corresponding to the G1‐phase (C2) and the G2‐phase (C4) nuclei. The x‐axis shows the intensity of total DNA fluorescence measured through a 585/42 nm band‐pass filter in arbitrary units, whereas the y‐axis indicates the number of observed nuclei. From this graph the genome size of tanoak is estimated to be 785 Mb.

## Slide 3
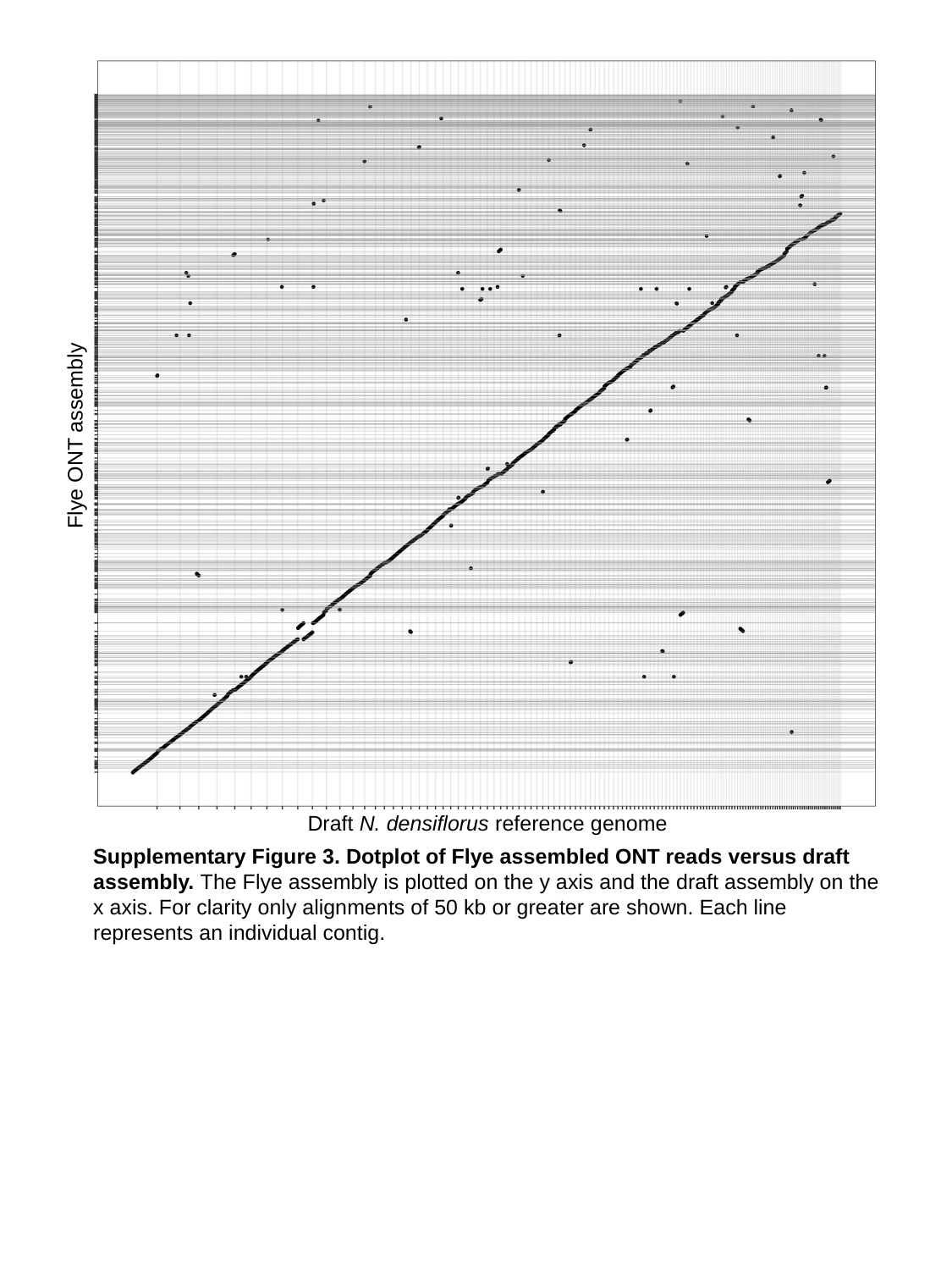

Flye ONT assembly
Draft N. densiflorus reference genome
Supplementary Figure 3. Dotplot of Flye assembled ONT reads versus draft assembly. The Flye assembly is plotted on the y axis and the draft assembly on the x axis. For clarity only alignments of 50 kb or greater are shown. Each line represents an individual contig.

## Slide 4
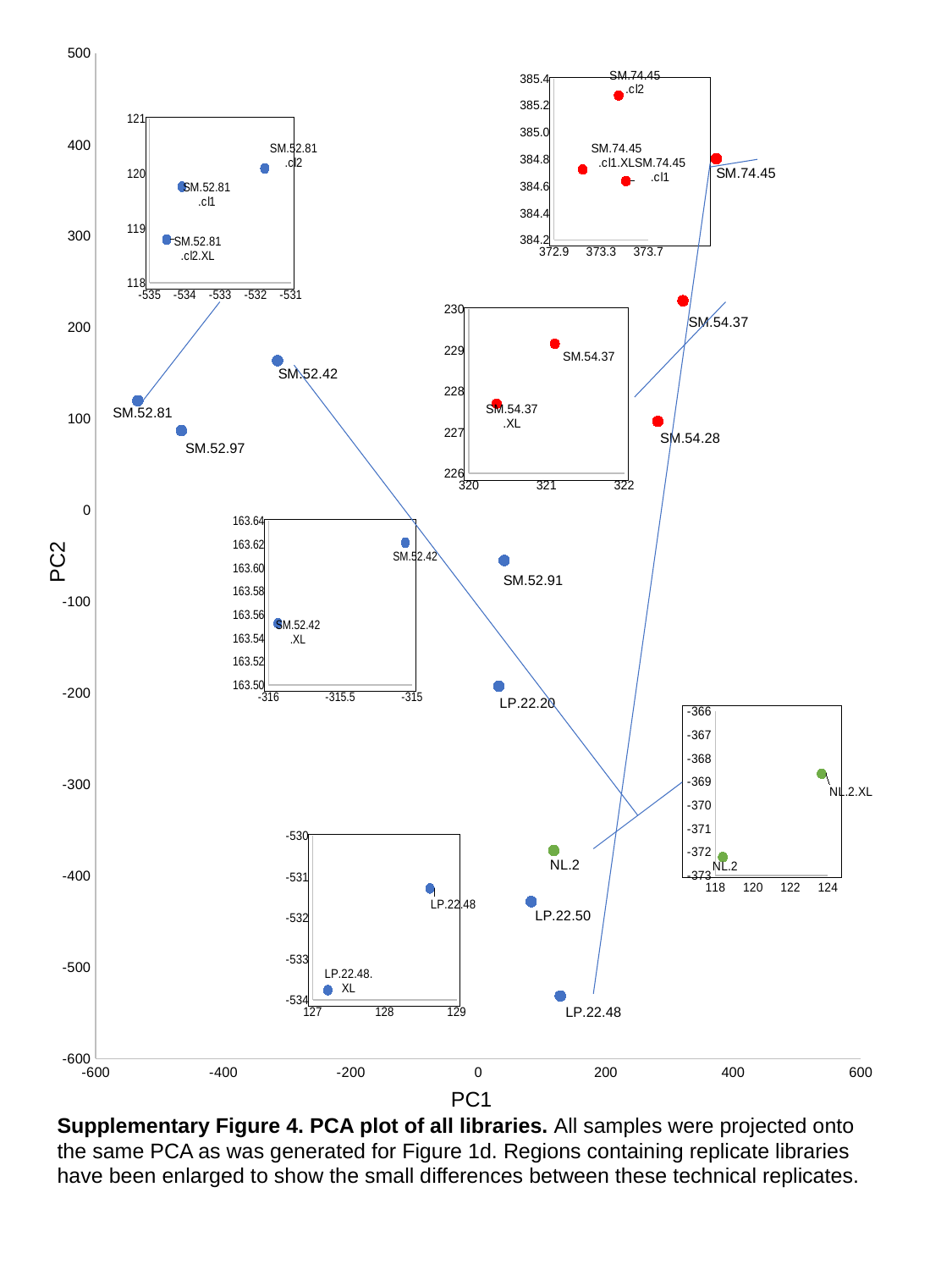

### Chart
| Category | SM.52.42 | SM.52.81 | SM.52.97 | SM.54.37 | LP.22.20 | LP.22.48 | LP.22.50 | NL.2 | SM.52.91 | SM.54.28 | SM.74.45 |
|---|---|---|---|---|---|---|---|---|---|---|---|
### Chart
| Category | SM.74.45.cl2 | SM.74.45.cl1 | SM.74.45.cl1.XL |
|---|---|---|---|
### Chart
| Category | SM.52.81.cl1 | SM.52.81.cl2 | SM.52.81.cl2.XL |
|---|---|---|---|
### Chart
| Category | SM.54.37 | SM.54.37.XL |
|---|---|---|
### Chart
| Category | SM.52.42 | SM.52.42.XL |
|---|---|---|PC2
### Chart
| Category | NL.2 | NL.2.XL |
|---|---|---|
### Chart
| Category | LP.22.48 | LP.22.48.XL |
|---|---|---|PC1
Supplementary Figure 4. PCA plot of all libraries. All samples were projected onto the same PCA as was generated for Figure 1d. Regions containing replicate libraries have been enlarged to show the small differences between these technical replicates.

## Slide 5
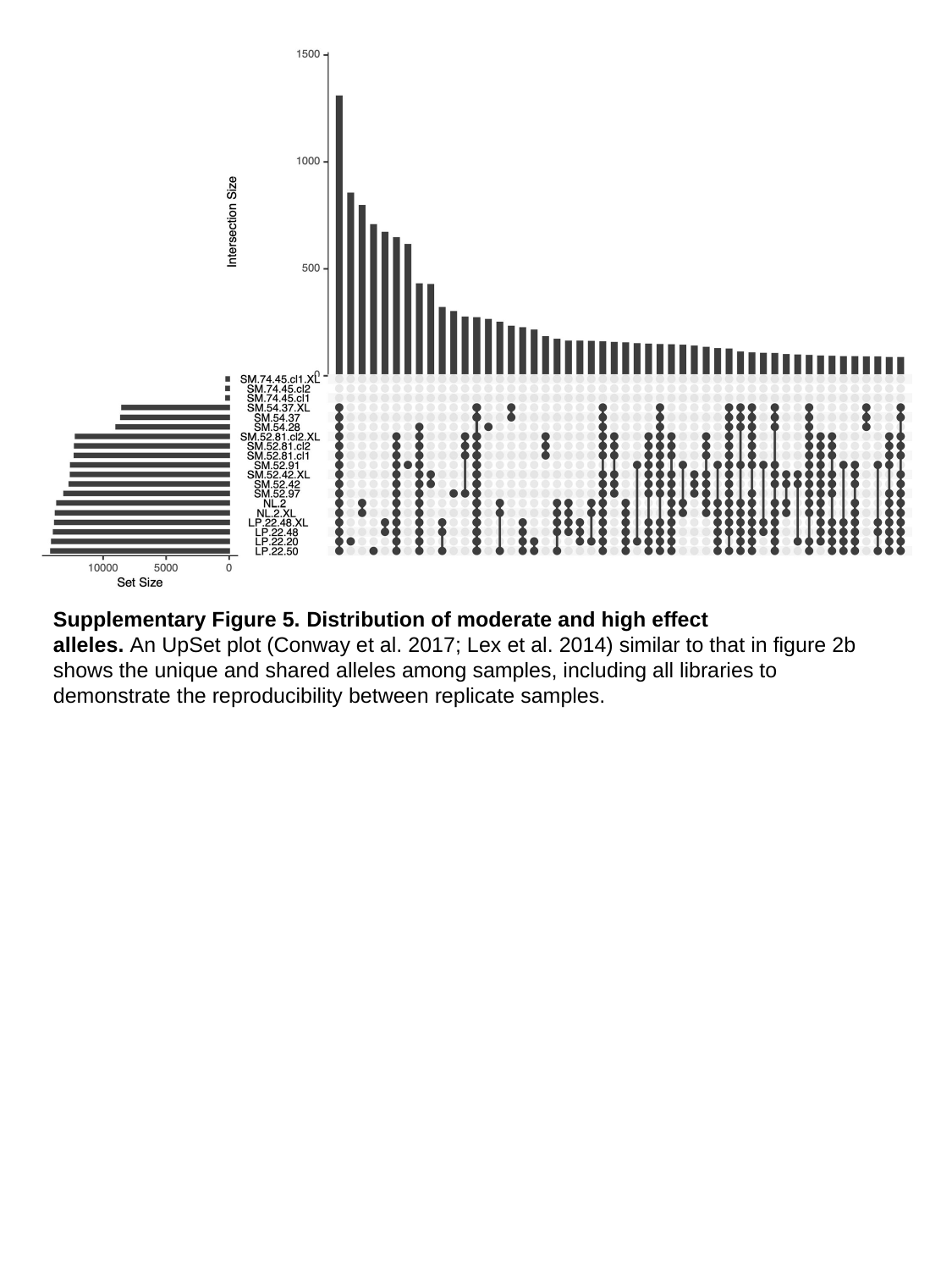

Supplementary Figure 5. Distribution of moderate and high effect alleles. An UpSet plot (Conway et al. 2017; Lex et al. 2014) similar to that in figure 2b shows the unique and shared alleles among samples, including all libraries to demonstrate the reproducibility between replicate samples.

## Slide 6
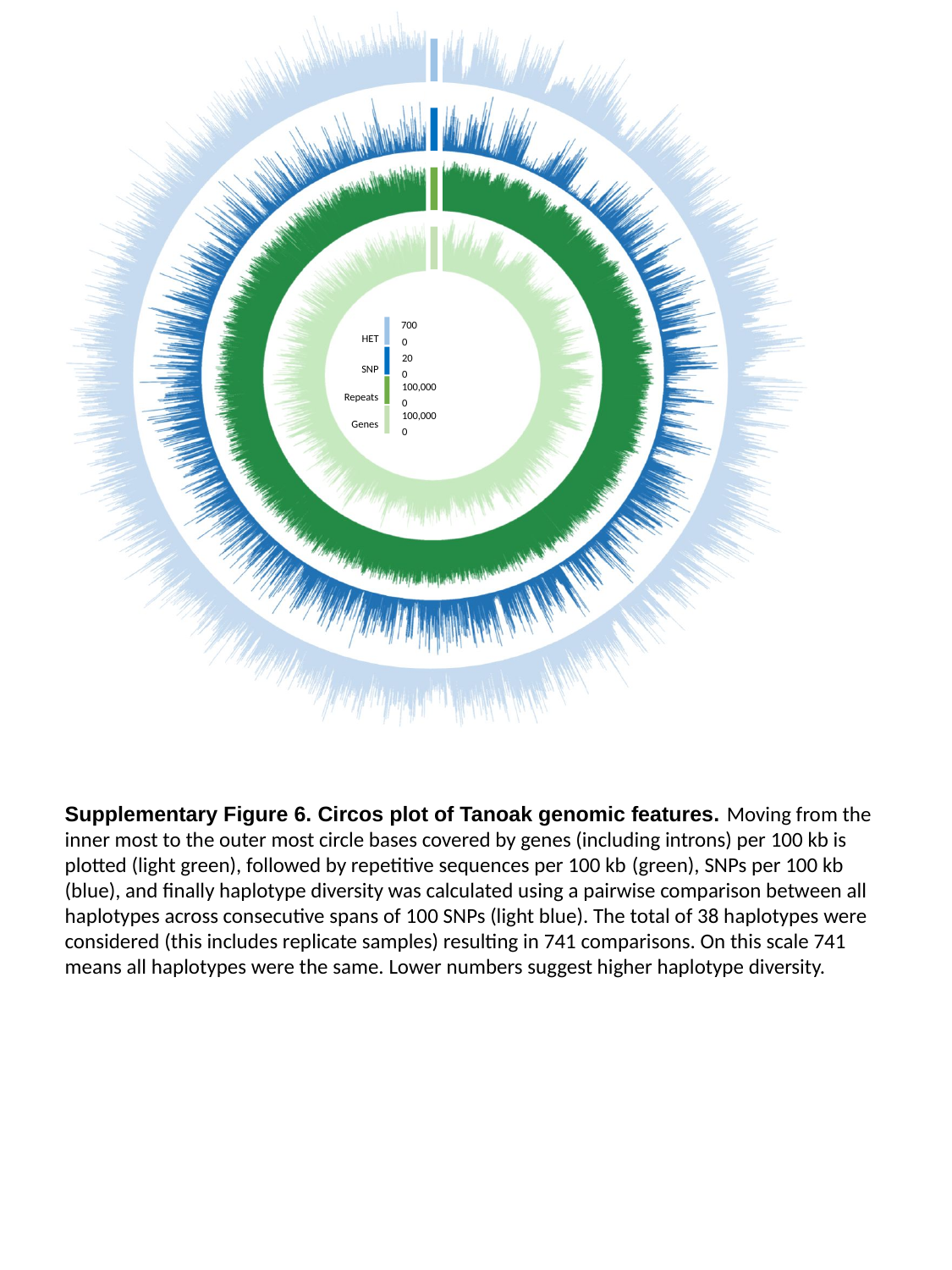

700
HET
0
20
SNP
0
100,000
Repeats
0
100,000
Genes
0
Supplementary Figure 6. Circos plot of Tanoak genomic features. Moving from the inner most to the outer most circle bases covered by genes (including introns) per 100 kb is plotted (light green), followed by repetitive sequences per 100 kb (green), SNPs per 100 kb (blue), and finally haplotype diversity was calculated using a pairwise comparison between all haplotypes across consecutive spans of 100 SNPs (light blue). The total of 38 haplotypes were considered (this includes replicate samples) resulting in 741 comparisons. On this scale 741 means all haplotypes were the same. Lower numbers suggest higher haplotype diversity.
